# Use of simulation as a needs assessment to develop a focused team leader training curriculum for resuscitation teams

**DOI:** 10.1186/s41077-020-00124-2

**Published:** 2020-05-27

**Authors:** Susan Coffey Zern, William J. Marshall, Patricia A. Shewokis, Michael T. Vest

**Affiliations:** 1 Virtual Education and Simulation Training (VEST) Center, Christiana Care Health System, 4755 Ogletown-Stanton Road, Ammon MEC LE86B, Newark, Delaware 19718 USA; 2 Nutrition Sciences Department, College of Nursing and Health Professions; School of Biomedical Engineering, Science and Health Systems, and Department of Teaching, Learning & Curriculum, School of Education, Drexel University, 3rd Floor, Room 382, Parkway Building, 1601 Cherry Street, Mail Stop 31030, Philadelphia, PA 19102 USA; 3 Department of Internal Medicine, Section of Pulmonary and Critical Care Medicine, Christiana Care Health System, 4755 Ogletown-Stanton Road, Medical Intensive Care Unit, 3E, Newark, Delaware 19713 USA; 4 Sidney Kimmel Medical College, Philadelphia, PA USA

**Keywords:** Cardiac Arrest, Medical Education, Advanced Cardiac Life Support, Team Training, curriculum development, needs assessment, gap analysis

## Abstract

**Background:**

Many inpatients experience cardiac arrest and mortality in this population is extremely high. Simulation is frequently used to train code teams with the goal of improving these outcomes. A key step in designing such a training curriculum is to perform a needs assessment. We report on the effectiveness of a simulation-based training program for residents designed using unannounced in-situ simulation cardiac arrest data as a needs assessment.

**Methods:**

In order to develop the curriculum for training, a needs assessment was done using in-situ simulation. Prior to instruction, residents were assessed in their ability to lead a simulated resuscitation using a standardized checklist. During the intervention phase, residents participated in didactic and team training. The didactic training consisted of pharmacology review, ACLS update and TeamSTEPPS training. Residents took turns as code team leader in three simulation sessions. Rapid cycle deliberate practice (RCDP) was employed as part of simulation sessions. All residents returned, for post-intervention assessment. Mean pre-post test scores were analyzed to determine if there was a significant difference.

**Results:**

Twenty-seven residents participated. Mean pre-training assessment score was 47.6 (95% CI 37.5-57.9). The mean post-training assessment score was 84.4 (95% CI 79.0-89.5). The mean time to defibrillation after pads were placed in scenario with shockable rhythm decreased from 102.2 seconds (95% CI 74.0-130.5) to 56.3 (95% CI 32.7-79.8).

**Conclusion:**

Using unannounced in-situ cardiac arrest simulations as a needs assessment, a simulation-based training program was developed that significantly improved resident performance as team leader. Future work is needed to determine if this improvement translates into patient benefits and is sustainable. However, in-situ simulation is a promising tool for curriculum development.

## Background

Cardiac arrest affects approximately 209,000 adult patients in hospitals in the United States every year [1]. Additionally, national statistics show outcomes are poor for those patients. Only 1 in 4 in hospital cardiac arrest patients will survive to hospital discharge. Moreover, those patients that survive often have neurological sequela from the event [2]. Survival for patients who have cardiac arrest at night and on weekends has been shown to be worse than for patients who undergo cardiac arrest during the work day using national data from both the United States and the United Kingdom [3, 4].

While it is typically a rare event for an individual healthcare provider, a cardiac arrest can be called multiple times a week in our 1,100-bed private teaching hospital system. Our inpatient code teams are led by a resident physician who has completed at least one post graduate year of training and American Heart Association (AHA) Advanced Cardiac Life Support (ACLS) training. These residents typically do not receive formal training in teamwork and communication or team leader training, yet they are expected to employ these nontechnical skills during a cardiac arrest event [5, 6]. Although the AHA has imbedded team leader training into the ACLS courses, the major focus of the course continues to be on medical management. Additionally, teamwork and communication skills necessary to be a team leader are not a formal component of the medical education curriculum for medical students or residents [7]. As a result of limited training, this skill set is difficult to employ when the resident is involved in an emergency situation with a high mental workload, new skills, and an ad hoc team [7, 8]. The AHA 2015 ACLS guideline updates recognize that ACLS training every two years is not sufficient [9]. However, while supporting the use of simulation training in general, they acknowledge that the optimal approach to ACLS education for resuscitation team members is unknown and call for additional research in this area.

Although resuscitation training is a common need, it is a complex undertaking for many institutions [10]. Curriculum development as described by Kern, needs to address multiple steps. One of the steps in developing the curriculum is determining the needs assessment. This step allows for an understanding of the differences between the learner’s expected performance versus their actual performance [11]. While traditionally simulation is used for training or for an intervention, it can also be used to determine gaps in knowledge, skill and abilities. Using in-situ simulation to perform a needs assessment can inform curriculum development targeted to a specific learner group. We hypothesized that simulation can be used as an effective tool for the purpose of developing a curriculum.

The goal of this study was to determine if a needs assessment performed using in-situ simulation would be an effective method for simulation-based resuscitation curriculum development, as measured by improved resident performance. We choose time to defibrillation as the primary performance outcome because it is easily measured, objective, and associated with clinically important outcomes.

## Methods

### Needs assessment

In order to accurately define the needs assessment for resuscitation curriculum development, we used in-situ simulation sessions. We conducted five unannounced in-situ cardiac arrest simulations throughout our hospital system. We specifically focused on the behavior of the team leader, as the curriculum would be designed to train only the team leader. Since our hospital resuscitation team is a contingency team the residents are a stable member and as such could have a substantial impact on overall team effectiveness.

In each of the in-situ simulations, a resident physician was the team leader. Team Leader behavior was observed by S.C.Z., W.M. and M.V. We evaluated each simulated in-situ cardiac arrest using ACLS guidelines and the TeamSTEPPS Team Performance Observation Tool [12]. Table 1 shows the areas of opportunity noted in at least four of five in-situ simulations. The areas of opportunity informed curriculum development for our intervention. Our intervention as described below was designed to ensure that the areas of opportunity noted during our needs assessment, would be taught and assessed.

**Table 1:** Results of in situ Evaluation Parameters for Needs Assessment

**TeamSTEPPS Performance Observation - Areas of Opportunity Noted**	
Team Leader did not Identify themselves as leading the resuscitation	
Team Leader did not assign roles and responsibilities	
Team leader failed to maintain situational awareness throughout the code	
Team Leader did not foster communication to ensure team members have a shared mental model	
Team Leader failed to collaborate with team members	
Team Leader did not provide timely and constructive feedback to team members, i.e. rate and quality of chest compressions	
Closed Loop Communication was lacking or non-existent during the resuscitation	
**AHA ACLS Guidelines – Areas of Opportunity Noted**	
Quality of chest compressions varied with team leader failing to monitor and address	
Airway management was not assessed	
Time to initial shock was variable	
Cardiac rhythm was not announced to the team	

AHA: American Heart Association, ACLS: Advanced Cardiac Life Support

### Study design

We conducted a pretest-posttest study to evaluate the impact of a new, annual resident resuscitation team leader training curriculum designed based on needs assessment using in-situ simulation. The course was conducted in the Virtual Education and Simulation Training (VEST) Center at the Christiana Care Health System. This study was submitted to the Christiana Care Institutional Review Board and was determined to be exempt.

### Participants

Participants were resident physicians completing their first post graduate year (PGY-1) of residency. They were required to be previously certified in Basic Life Support (BLS) and ACLS. All PGY-1 residents in internal and family medicine programs in the spring of 2016 at Christiana Care were required to participate in the training.

### Curriculum description and intervention

The curriculum started with a pre-assessment of the resident’s skills in the simulation center. This was done after curriculum was set and was separate from the in-situ simulations used for the needs assessment. Each resident was assessed on their ability to lead a cardiac arrest with a standardized interprofessional team. A team of confederates was trained to play the roles of a respiratory care provider, a medical intensive care nurse, a bedside nurse and three medical students using a standardized scenario. The scenario’s objectives and checklist evaluation were based on the gaps noted in the in-situ needs assessment. In each scenario, two errors were purposely made by the standardized team; one was inaccurate closed loop communication by the MICU nurse and the other was a medical student slowing down the rate of chest compressions. METIman® pre-hospital model or nursing model, patient simulator (CAE Healthcare) was used. The resident was blinded to the areas of assessment as the team leader. The session was terminated at the end of the third cycle of chest compressions. Each pre-assessment was video recorded and evaluated using a checklist, by one of two trained evaluators. After running the cardiac arrest, each resident was given a rhythm recognition test using HEARTSIM 200, rhythm generator (Laerdal) where they had 20 seconds to report each rhythm to an evaluator.

**Fig. 1:**
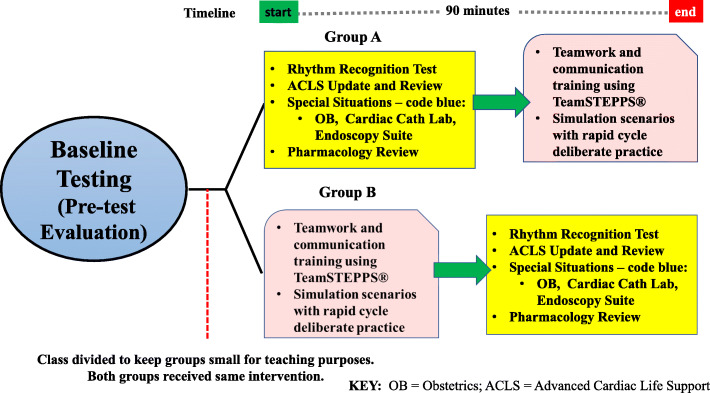
Class divided to keep groups small for teaching purposes. Both groups received same intervention

For the intervention, all the residents received the same training including both didactic and TeamSTEPPS® simulation training. The residents were split into two groups (Group A and Group B) specifically to ensure that the groups were small in size, not to assess order of the training. Figure 1 outlines the agenda. One group attended didactic lectures for 1½ hours which included pharmacology review, ACLS update and review, and special situations (i.e., caring for coding pregnant patients). They were given an electronic multiple-choice test to assess their knowledge of the content (content exam).

The other group attended TeamSTEPPS® didactic training session by a TeamSTEPPS® master trainer, focusing on the role of the team leader. They then participated in three simulation scenarios with rapid cycle deliberate practice for 1 ½ hours. TeamSTEPPS® is an evidence-based systematic training, developed by the Department of Defense (DOD) and the Agency for Healthcare Research and Quality (AHRQ), used to improve and integrate teamwork and communication into healthcare delivery. There are five tenets of TeamSTEPPS®; team structure, communication, leading teams, situational monitoring and mutual support [13]. These key principles are important for a resident to understand, exhibit and support as the resuscitation team leader.

After the TeamSTEPPS® didactic session, the residents then took turns being the team leader in three simulation scenarios (Table 2) with a standardized resuscitation team of trained confederates assuming the roles of the typical members who would normally respond, RCP, MICU Nurse, Bedside nurse, and three people to provide chest compression. The focus of the simulation scenarios was teamwork and communication. Rapid cycle deliberate practice (RCDP) was employed as part of the session with the faculty facilitators stopping the session for skills that required immediate correction, further practice or opportunities to highlight something done exceptionally well, as was described in Hunt, et al 2014 [14]. Only one resident at a time was the team leader, the other residents were in the room watching them lead the code but were not permitted to speak or assist in anyway. Each simulation session was performed in a different room with a different patient case. Each resident participated as the leader in one of the three scenarios. The resident led a cardiac arrest scenario and received immediate correction or praise for their performance. We chose RCDP as a means of debriefing to ensure that the residents were able to practice the new skills many times and learn vicariously while watching their peers.

**Table 2:** Team Work and Communication Scenarios using Rapid Cycle Deliberate Practice (RCDP) Debriefing

Case	Rhythm	Scenario
1	PEA Arrest	Adult patient was admitted overnight for deep tissue infection on left leg. He recently had a subclavian central line inserted. Breath sounds are decreased on right side of the chest one minute into the code. • Cardiac Arrest starts with bedside nurse in the room doing compressions • Code Team comes in with the resident team leader • After two minutes the confederate Respiratory Care Provider comments that ventilating has gotten more difficult
2	Slow V Tach	Adult patient is admitted for lumbar discectomy. He has peripheral IV access. • Cardiac arrest starts with multiple nurses in the room • Pads are on the chest • CPR is in progress • Defibrillator is in AED mode and is still on • Code team comes in with the team leader
3	PEA Arrest	Adult patient was admitted overnight with concern for sepsis. He has peripheral IV access. Patient was noted to have elevated lactate levels as per bedside nurse report. • Cardiac arrest starts with bedside nurse in the room • No pads are on the patient • Code team comes in with the team leader • After the first rhythm check the MICU nurse states that the IV is lost and not working

PEA – Pulseless Electrical Activity, V Tach – ventricular tachycardia, CPR – cardiopulmonary resuscitation, AED – automatic electrical defibrillator, IV – intravenous line

All residents individually returned to the simulation center between three to five weeks later for a post-intervention assessment. The same scenario used for the pre-assessment was used again. The team leader was expected to run the cardiac arrest with a standardized interprofessional team, and the standardized scenario. Each assessment was video recorded, evaluated by checklist by one of two trained evaluators using the same checklist as the pre-assessment. The resident was debriefed after the cardiac arrest. Overall scores were compared pre and post training.

### Statistical analysis

Descriptive statistics and assumptions for parametric tests were calculated for the following variables cardiac arrest pre-test and post-test scores; rhythm visible-time to shock delivered (RVTSD) pre-test and post-test scores; content exam and rhythm recognition test. If the normality assumptions were violated, then appropriate non-parametric tests were calculated. To determine the effectiveness of the intervention, parametric paired t-tests were used to compare pre and post test scores on the code blue and RVTSD tests. The content exam (electronic multiple choice test) and rhythm tests were administered only once and a criterion of 80% competency or better was used for passing. A one sample t-test was calculated for the content exam and rhythm test. Effect sizes were calculated and used to aid in interpretation of the data. The effect size index for the paired t-test and one-sample t-test is Cohen’s d_z._ [15]. Cohen’s d_z_ is interpreted as d_z_ = 0.20, 0.50 and > 0.80 as small, medium and large effects, respectively. To assess any order effects of the grouping of participants, we calculated independent samples t-tests (two-tailed) with a significance criterion of α = 0.05. Since there were multiple tests employed, we used a Bonferroni adjustment to control for Type I error inflation (alpha/6 = 0.0083). The significance criterion for all tests was set at α= 0.05. Number Cruncher Statistical Software (NCSS ver. 9; www.ncss.com) was used for the analyses.

## Results

A total of 27 residents completed the training; 14 were female and 13 male, there were internal medicine (n=12), combined internal medicine/pediatrics (n= 4) family medicine (n=6) and there were combined internal medicine/emergency medicine residents or family medicine/emergency medicine (n=5). Sixty-one percent reported having attended five or more cardiac arrests, in clinical care within the past year.

The competency level was set at 80% for the all assessments. An 80% or better performance is typically noted as the criterion for competent performance [16]. Descriptive statistics and 95% confidence intervals are reported in Table 3 for all measures. The effect of simulation training with rapid cycle deliberate practice significantly improved cardiac arrest performance overall [t(26) = -6.248, p< 0.001, d_z_ = -1.20] and time to shock delivery (RVTSD) [t(26) = 3.127, p =0.004, dz = 0.60) specifically. Cardiac arrest scores showed a large effect while RVTSD scores resulted in a moderate-to-large effect. A significant effect was detected for the mean content exam [t(26) = 2.93, p = 0.003, d_z_ = 0.56] indicating that the content exam (electronic multiple choice test) was able to reliably discriminate between high performing residents and low performing residents. No significant difference was detected for the rhythm test [t(26) = 0.132, p 0.448, d_z_ = 0.03] showing that there was no reliable discrimination between the high performing and low performing residents based on the rhythm test. There was no difference in performance related to whether residents were assigned to group A or group B (p>0.05).

**Table 3:** Descriptive Statistics and 95% Confidence Intervals of the Dependent Measures

Variable	Time	Mean + SD	95% Confidence Interval (LL, UL)
Cardiac arrest team leader performance	Pre-test	47.6 + 25.9	(37.3, 57.9)
Cardiac arrest team leader performance	Post-test	84.3 + 13.3	(79.0, 89.5)
RVTSD	Pre-test	102.2 + 21.4	(74.0, 130.5)
RVTSD	Post-test	56.3 + 59.5	(32.7, 79.8)
Rhythm Test	Once	80.4 + 14.5	(74.6, 86.1)
Content Exam	Once	86.0 + 10.7	(81.8, 90.3)

RVTSD – rhythm visible time to shock delivered, SD – standard deviation, LL – lower limit, UL – upper limit. Content Exam was an electronic multiple choice exam

In the debriefing, the residents commented that in the pre assessment the confederate team was difficult to lead and seemed to lack content knowledge and skill in resuscitation. Their comments at the post assessment were entirely different; they felt that the team was much better trained and more knowledgeable regarding resuscitation. They initially did not attribute this to their improved leadership skills. However, we used the same simulation case and standardized team of confederates for pre and post assessments so that we controlled the team’s performance and ensured that it was not significantly different between the two assessments.

## Discussion

The use of in-situ simulation to determine a needs assessment enabled the simulation curriculum to incorporate the exact problems and barriers the resident would experience in clinical care. It ensured that we were teaching to the actual gaps in knowledge and skills. We addressed issues that the residents demonstrated during the gap analysis such as closed loop communication, maintaining situational awareness and assigning roles and responsibilities. Importantly, after training in these leadership skills we observed an improvement in the clinically important outcome of time to defibrillation.

Needs assessments in medical education are often done using data gathered from surveys, structured interviews, observations of clinical practice, or peer review data [17]. Focus groups have been described as a method to assess needs for simulation based emergency training [18]. The incorporation of input from multiple stakeholders (insurers, educational institutions, funders, employers, regulators) has also been described in the development of simulation curriculum [19]. In addition to the above methods for gathering data to inform curriculum development, in-situ simulation can be considered an additional option. To our knowledge, this is the first time that using simulation to perform both a needs assessment and an intervention has been reported.

By using in-situ simulation as a needs assessment tool, we developed a focused curriculum to meet the needs of our residents, rather than just repeating training in ACLS. While AHA acknowledges that every 2 years training in ACLS is not sufficient, the best approach to training during that interim 2-year period is unknown. We believe that in-situ simulation alone is unlikely to correct deficits. However, using in-situ simulation to identify gaps and addressing them with a focused curriculum informed by this assessment, has potential to be an effective approach. We are currently using in-situ simulation for needs assessment for other areas of emergency response including pediatric, surgical, neurologic and obstetric emergencies. Future work will be needed to determine the optimal time frame to repeat in-situ simulation for the purpose of re-assessing the need for curriculum developed in this fashion.

We found that training of the team leader in ACLS and teamwork and communication skills using TeamSTEPPS® with RCDP can improve team performance in simulated cardiac arrest. Prior work has shown improvements in clinical outcomes of pediatric patients after simulation based code team training [10]. Our study differed in that we focused our intervention only on the resident code team leaders. This is important because many institutions, including ours, are faced with training thousands of healthcare providers to respond to cardiac arrest emergencies. While we believe that training the actual responding team is optimal, when this is not logistically possible, our data suggest that training focused on the team leader may still have a positive impact.

We noted that many cycles of deliberate practice were needed for residents to effectively employ TeamSTEPPS® skills. Using simulation scenarios with RCDP allowed the residents to practice the expected technical and non-technical skills over and over until they became a habit. Our goal was to make sure the newly learned TeamSTEPPS® concepts and tools would be solidly incorporated into each resident’s repertoire, i.e. assessing chest compressions, assigning roles and responsibilities, etc. RCDP may be particularly effective debriefing method to use in a focused simulation developed using in-situ simulation as a needs assessment tool.

Our study has several limitations. First, we cannot determine the impact our training had on actual patients. Second, while all post-graduate year one medicine residents at our institution participated, this was a small study at one institution, and it would be important to know if the same findings could be replicated in larger studies at other institutions. Also, all participants received training, so the lack of a control group is a limitation of this study. Future work will need to determine the durability of this training and impact on actual patients. Lastly, the training sequence may have had an impact on resident learning and retention.

## Conclusions

In conclusion, a novel code team leader training course using in-situ simulation data to develop the curriculum and combining TeamSTEPPS® principals and ACLS science update can provide sustained improvement in resident performance as code team leaders.

## Data Availability

The datasets generated and/or analyzed during the current study are not publicly available due to need to protect the privacy of the trainees who participated but are available from the corresponding author on reasonable request.

## Abbreviations

ACLS: Advanced Cardiac Life Support RCDP Rapid Cycle Deliberate Practice AHA American Heart Association TeamSTEPPS Team Strategies and Tools to Enhance Performance and Patient Safety BLS Basic Life Support MICU Medical intensive care unit RCP Respiratory care provider DOD Department of Defense AHRQ Agency for Healthcare Research and Quality RVTSD Rhythm Visible-time to shock delivered

## References

[1] Kronick SL, Kurz MC, Lin S (2015). Part 4: Systems of Care and Continuous Quality Improvement: 2015 American Heart Association Guidelines Update for Cardiopulmonary Resuscitation and Emergency Cardiovascular Care. Circulation..

[2] Barbeito A, Bonifacio A, Holtschneider M (2015). In situ simulated cardiac arrest exercises to detect system vulnerabilities. Simul Healthc.

[3] Peberdy MA, Ornato JP, Larkin GL (2008). Survival from in-hospital cardiac arrest during nights and weekends. JAMA..

[4] Robinson EJ, Smith GB, Power GS, et al. Risk-adjusted survival for adults following in-hospital cardiac arrest by day of week and time of day: observational cohort study. BMJ Qual Saf. 2015. 10.1136/bmjqs-2015-004223 PMC5136724 26658774

[5] Blackwood J, Duff JP, Nettel-Aguirre A, Djogovic D, Joynt C (2014). Does teaching crisis resource management skills improve resuscitation performance in pediatric residents?*. Pediatr Crit Care Med.

[6] Fernandez Castelao E, Boos M, Ringer C, Eich C, Russo SG (2015). Effect of CRM team leader training on team performance and leadership behavior in simulated cardiac arrest scenarios: a prospective, randomized, controlled study. BMC Med Educ.

[7] Burden AR, Pukenas EW, Deal ER (2014). Using Simulation Education With Deliberate Practice to Teach Leadership and Resource Management Skills to Senior Resident Code Leaders. J Grad Med Educ.

[8] Wright MC, Taekman JM, Endsley MR (2004). Objective measures of situation awareness in a simulated medical environment. Qual Saf Health Care.

[9] Bhanji F, Donoghue AJ, Wolff MS (2015). Part 14: Education: 2015 American Heart Association Guidelines Update for Cardiopulmonary Resuscitation and Emergency Cardiovascular Care. Circulation..

[10] Knight LJ, Gabhart JM, Earnest KS, Leong KM, Anglemyer A, Franzon D (2014). Improving code team performance and survival outcomes: implementation of pediatric resuscitation team training. Crit Care Med.

[11] Kern DE (1998). Curriculum development for medical education : a six step approach.

[12] Team Performance Observation Tool. https://www.ahrq.gov/teamstepps/longtermcare/sitetools/tmpot.html, 2014.

[13] TeamSTEPPS 2.0. http://www.ahrq.gov/teamstepps/instructor/index.html. Accessed November, 2018.

[14] Hunt EA, Duval-Arnould JM, Nelson-McMillan KL (2014). Pediatric resident resuscitation skills improve after “rapid cycle deliberate practice” training. Resuscitation..

[15] Lakens D (2013). Calculating and reporting effect sizes to facilitate cumulative science: a practical primer for t-tests and ANOVAs. Front Psychol.

[16] Wass V, Wakeford R, Neighbour R, Van der Vleuten C (2003). Royal College of General P. Achieving acceptable reliability in oral examinations: an analysis of the Royal College of General Practitioners membership examination's oral component. Med Educ.

[17] Grant J (2002). Learning needs assessment: assessing the need. BMJ..

[18] Wehbi NK, Wani R, Yang Y (2018). A needs assessment for simulation-based training of emergency medical providers in Nebraska, USA. Adv Simul (Lond).

[19] Edwards S, Tuttle N (2019). Using stakeholder input to inform scenario content: an example from physiotherapy. Adv Simul (Lond).

